# Expression Analysis and Functional Validation of DcTPSb1 in Terpene Synthesis of *Dendrobium chrysotoxum*

**DOI:** 10.3390/cimb47010025

**Published:** 2025-01-03

**Authors:** Yuxuan Jin, Shuting Zhou, Zhihui Du, Weize Wang, Zhilin Chen

**Affiliations:** 1 Guizhou Horticulture Institute/Horticultural Engineering Technology Research Center of Guizhou, Guizhou Academy of Agricultural Sciences, Guiyang 550000, China; jinyuxuan@whu.edu.cn (Y.J.); dzh8928088@163.com (Z.D.); wangweize_gz@163.com (W.W.); 2 Natural Products Research Center of Guizhou Province, Guiyang 550000, China; zstaipt0117@163.com

**Keywords:** *Dendrobium chrysotoxum*, *DcTPSb1*, terpene synthase, floral fragrance

## Abstract

Terpenes are critical components of the floral fragrance component in *Dendrobium chrysotoxum*, synthesized by terpene synthase (TPS). Analysis of the *D. chrysotoxum* genome and transcriptional data revealed that the gene *DcTPSb1* was significantly up-regulated during flowering periods, showing a strong correlation with the accumulation of aromatic monoterpenes in the floral components of *Dendrobium chrysotoxum*. Consequently, the *DcTPSb1* gene was selected for further analysis. *DcTPSb1* exhibited elevated expression levels in flowers among four organs (roots, stems, leaves, flowers) of *D. chrysotoxum*, with the highest expression observed during the blooming phase, which aligned with the accumulation of volatile terpenes during flowering. DcTPSb1, located in the chloroplasts, was identified as a member of the TPS-b subfamily associated with monoterpenes synthesis, showing close phylogenetic relationships with homologous proteins in related plant species. An analysis of the promoter region of *DcTPSb1* indicated that it may be regulated by methyl jasmonate (MeJA) responsiveness. Functionally, DcTPSb1 was shown to catalyze the conversion of geranyl diphosphate (GPP) to linalool, ocimene, and (-)-α-pinitol in vitro. Overexpression of *DcTPSb1* in tobacco resulted in a significant increase in terpenoid release during the blooming stage; however, the up-regulated substances did not include their catalytic products. The classification of DcTPSb1 as a terpene synthase capable of producing multiple products provides valuable insights into the complex biosynthesis of terpenes in orchids. These findings enhance our understanding of the functional diversity of *DcTPSb1* and the processes involved in terpene biosynthesis in orchids.

## 1. Introduction

The floral fragrance of plants serves multiple functions, including the attraction of pollinators, the provision of resistance to physical threats, and the defense against pests, herbivores, and pathogens [1]. Typically, floral fragrances consist of a range of low-molecular-weight secondary metabolic volatiles, with terpenes, aliphatic compounds, benzene ring/phenylpropane compounds, sulfur compounds, and nitrogen-containing compounds constituting the primary components [2]. Among these, terpenoids are particularly significant in the floral aroma of many plant species due to their structural diversity and widespread occurrence across various organisms. Notably, terpenoids play a crucial role in the floral fragrance of *Dendrobium* species, including *D. loddigesii*, *D. chrysotoxum*, *D. officinale*, and *D. hancockii* [3,4,5,6,7].

The synthesis of terpenoids involves two interconnected yet distinct pathways: the mevalonate (MVA) pathway in the cytoplasm and the 2-C-methyl-D-erythritol-4-phosphate (MEP) pathway in the plastid [8,9]. These pathways produce the structural units isopentenyl pyrophosphate (IPP) and dimethyl allyl pyrophosphate (DMAPP). The C5 precursors, DMAPP and IPP, subsequently lead to the formation of direct precursors like farnesyl diphosphate (FPP), geranyl diphosphate (GPP), and geranylgeranyl diphosphate (GGPP). In the cytosol, FPP is converted to sesquiterpene via the first pathway, in contrast, the second pathway that occurs in the plastid leads to the conversion of GPP to monoterpenes and GGPP to diterpenes [10]. At the branching point of the isoprenoid pathway, TPS plays a crucial role in influencing the diversity of plant terpenoids. The types and activities of TPS within the TPS family are essential for the synthesis of aromatic terpenes in fragrant-flowered plants. The type and expression of TPS genes are vital in the biosynthesis and accumulation of terpenoids, serving as significant regulators of plant floral fragrance [1,4]. While several TPSs responsible for aromatic terpene synthesis have been identified in ornamental plants such as *Lilium* [11], *Jasminum* [12], *Cymbidium* [13], and *Phalaenopsis* [14], only *Dendrobium officinale* within the genus *Dendrobium* has been extensively studied. Zhao et al. [15] identified an important candidate gene for the monoterpenoid synthase geraniol synthase (GES), *DoGES1*, and verified its function through prokaryotic expression and transgenic experiments in a model plant. Subsequently, Li et al. (2021) associated 13 *DoTPS* genes with terpenoid floral aroma compounds through transcriptional and metabolic joint analysis of two different cultivated varieties of *Dendrobium officinale*. Yu et al. concluded that DoTPS10 functions as a single-product enzyme (converting GPP to linalool), which can be upregulated by DobHLH4 [16,17]. However, the functions and mechanisms of action of TPS in *Dendrobium Sw.* remain to be elucidated.

The assembly and release of the chromosomal-level genome of *Dendrobium chrysotoxum* have facilitated improved material selection for investigating the mechanism underlying aroma synthesis in this fragrant species [18]. Through a combined analysis of transcriptional metabolism, we examined the composition and dynamic changes in the floral aroma in *Dendrobium chrysotoxum*, elucidating the critical role of aromatic terpenoids in the formation of its floral characteristics. As the density of terpenes accumulates during the flowering period, the expression of certain genes is upregulated. Then, three TPSb genes (*DcTPSb1*, *DcTPSb3*, and *DcTPSb4*) that exhibited a strong correlation with terpene compounds at bloom were identified [4,19]. Of the three, *DcTPSb1* demonstrated the highest expression level in RNA-seq across the three flowering periods, showing a significant correlation with the accumulation of seven representative aromatic monoterpenes in the floral components of *Dendrobium chrysotoxum*. The seven representative aromatic monoterpenes mentioned above included 4-methylene-1-(1-methylethyl)-Bicyclo [3.1.0]hex-2-ene; (-)-trans-Isopiperitenol; 2,6,10,10-tetramethyl-1-Oxaspiro [4.5]dec-6-ene; E,E-2,6-Dimethyl-1,3,5,7-octatetraene; 2-methyl-6-methylene-1,7-Octadiene; 1,5,5,6-tetramethyl-1,3-Cyclohexadiene; Isopinocarveol. However, the function of *DcTPSb1* has yet to be verified, and the specific aromatic terpenoid products, along with the transcriptional regulatory mechanism during flowering, remain to be elucidated. This study aims to perform sequence analysis and functional validation of the floral fragrance gene *DcTPSb1* from *D. chrysotoxum*, thereby providing a theoretical foundation for understanding the development of aromatic characteristics in ornamental *Dendrobiums*. Additionally, the findings of this research may serve as valuable genetic resources for the breeding of aromatic *Dendrobiums*.

## 2. Materials and Methods

### 2.1. Plant Materials

Plants of *D. chrysotoxum* were originally sourced from Yunnan province and then transplanted to the greenhouse of the Institute of Horticulture, Guizhou Academy of Agricultural Sciences. The growth conditions were maintained under natural light with a temperature range of 20–25 degrees Celsius. Samples of *D. chrysotoxum* were collected from the roots, stems, leaves, and flowers at various stages of anthesis, including bud, semi-open, and fully open stages, with three replicates for each stage. The collected plant samples were preserved at −80 °C. In parallel, *Nicotiana benthamiana* plants utilized for subcellular localization studies were cultivated in a growth room under controlled conditions at 22 °C with a photoperiod of 16 h.

### 2.2. Gene Cloning, Sequence Alignment, and Phylogenetic Analysis of the DcTPSb1

Total RNA was isolated from *D. chrysotoxum* tissues using Trizol (Invitrogen, Waltham, MA, USA) according to the protocol. Subsequently, the cDNA was synthesized with 800 ng of RNA in a 20 uL reaction system using the TUREscript 1st Stand cDNA SYNTHESIS Kit (Aidlab, Beijing, China) in adherence to the manufacturer’s instructions. The coding sequence of *DcTPSb1* was cloned with high fidelity using KOD FX polymerase (TOYOBO, Japan). The PCR program consisted of an initial incubation at 94 °C for 2 min, followed by 32 cycles of denaturation at 98 °C for 10 s, annealing at 55 °C for 30 s, and extension at 68 °C for 2 min, concluding with a final extension at 68 °C for 5 min. The length of the PCR product is illustrated in Figure S1. Simultaneously, Sanger sequencing was used to verify the sequences obtained. Afterward, the amino acid sequences of DcTPSb1 were subjected to multiple sequence alignment using Clustal X 2.0 [20], and an image was generated using ESPript 3.0 [21]. Subsequent to this, evolutionary analysis of the amino acid sequence of DcTPSb1 was performed using the neighbor-joining method in MEGA 11.0 software [22]. Finally, the secondary structure of the DcTPSb1 protein was elucidated through the SOPMA program [23].

### 2.3. Subcellular Location Prediction and Validation of DcTPSb1

Prediction tools such as WoLF PSORT [24] and PLoc-mPlant [25], which exhibit an accuracy exceeding 70%, were employed to predict the subcellular localization of the DcTPSb1 protein. The 1794 bp coding sequence of *DcTPSb1*, as presented in Table S1, did not contain the stop codon, was initially amplified, and then ligated into a modified pc1300 vector, which was regulated by the CaMV 35S promoter. Following this, 4-week-old tobacco plants with good growth status were selected, and the recombinant DcTPSb1-GFP plasmid bacterial solutions were delivered into the lower epidermis of tobacco leaves via injection with the aid of a syringe in 1 mL size without the nozzle. Then, experimental plants were appropriately labeled and incubated for 2 days under low light conditions. Labeled tobacco leaves injected with Agrobacterium were then excised, prepared as glass slides, and observed under a laser confocal microscope, with photographic documentation. During this experiment, fluorescence emissions from GFP and Chloroplast were detected using lasers with wavelengths of 488 nm and 640 nm, respectively, within the observation field of the FV1000 microscope (OLYMPUS).

### 2.4. Real-Time Quantitative PCR Analysis

To analyze the expression patterns of the *DcTPSb1* gene, quantitative reverse transcription–polymerase chain reaction(qRT-PCR) was conducted in different tissues of *D. chrysotoxum*. The qRT-PCR reaction was set up with a total volume of 10 µL, which included 5 µL of PCR Mix, 0.5 µL of both forward and reverse primers, 1 µL of cDNA, and the remaining volume was adjusted with water. The qRT-PCR program was set: at 95 °C for 3 min, 95 °C for 10 s, and 60 °C for 30 s in 39 cycles, with three biological and technical replicates. The amplification was conducted utilizing the BIO-RAD CFX Connect Real-Time System, in accordance with protocols. The relative abundance of 18 S served as the internal standard for quantification, calculated through the 2^−∆∆CT^ method [26]. Specific primer details can be found in Table S2.

### 2.5. Preliminary Parse of Cis-Acting Elements Inside DcTPSb1’s Promoter Area

The sequence region to be analyzed located 2000 bp in front of the translation initiation codon for *DcTPSb1* in *D. chrysotoxum* was retrieved from the *D. chrysotoxum* genome [18]. Subsequently, the PlantCARE online software was used to analyze potential cis-regulatory elements within the promoter region [27].

### 2.6. Prokaryotic Expression and Purification of DcTPSb1 Protein

Following the optimization of codons, the *DcTPSb1* gene was synthesized and ligated into the pDEST15 plasmid, which contained a glutathione S-transferase (GST) tag of 26 KDa. The recombinant plasmid, designated pDEST15-DcTPSb1, was then transformed into E. coli BL21 (DE3) competent cells. Positive clones were identified and incubated at 37 °C in the presence of ampicillin at OD600 nm of 0.6. The mixed cultures were obtained by 20 min centrifugation after a culture mixture was mixed with the inducing compound, Isopropyl-β-d-thiogalactopyranoside (IPTG), to reach a final concentration of 0.2 mM. Induction of protein expression was carried out by shaking the cultures at 180 rpm for 18 h at a temperature of 20 degrees Celsius. Once the fusion protein with expression and good water solubility was identified using SDS-PAGE, the purification process commenced. The purification began with high-pressure or ultrasonic crushing of the mixture, resulting in the obtaining of supernatant through centrifugation and subsequent filtration to remove impurities. The supernatant containing the target protein was gradually introduced into a purification column, allowing for slow dripping and collection, with this operation repeated approximately 3 times to maximize protein adsorption onto the beads. The column was rinsed around 6 times with a rinsing solution consisting of 50 mM Tris, pH 7.6. Subsequently, the elution buffer (50 mM Tris, 50 mM NaCl, 20 mM reduced glutathione, pH 8.0) was employed to elute the precipitate for further purification. The purified GST-DcTPSb1 protein was then subjected to additional analysis to verify its size and location.

### 2.7. Functional Verification of DcTPSb1 by Enzyme Assay In Vitro

The reaction mixture was composed of 2 ng of DcTPSb1 protein, HEPES (4-(2-Hydroxyerhyl) piperazine-1-erhanesulfonic acid) buffer (pH 7.2), 50 mM MgCl2, glycerol, 0.1 mM DTT (Dithiothreitol), and Geranyl pyrophosphate (GPP) as the substrate. Subsequently, the mixtures were incubated at 30 °C for 2 h in a total volume of 100 µL. The control group was added with high-temperature inactivated TPS protein, while other conditions were maintained identical to those of the experimental group. After the reaction was complete, an equal volume of n-hexane, which serves as an extractant, was added to the mixtures, which were vortexed for 2 min to ensure uniformity. After 10 min, the upper organic phase was extracted for analysis using gas chromatography–mass spectrometry (GC-MS). The ions detected were compared with the National Institute of Standards and Technology (NIST) database, allowing for the identification of matched substances as terpenes, which were determined to be the reaction products.

### 2.8. Detection of Volatile Metabolome in DcTPSb1 Overexpressing N. benthamiana Using GC-MS

The modified pC1300 vector was used to ligate the full-length coding sequence of *DoTPSb1*. Subsequent to this, a plasmid for plant invasion was generated by transforming Agrobacterium tumefaciens GV3101, which contained a target gene validated by sequencing, a 35S promoter, and conferred with hygromycin resistance. Using the leaf disk transformation system, Agrobacterium plasmid transfection was performed on the leaves of wild-type (WT) tobacco sterile tissue cultured seedlings. PCR experiments and GUS (β-glucuronidase) staining were conducted to identify the overexpressed (OE) plants (Figure S5). The latter followed the instructions provided in the kit (Huayueyang, Beijing, China). Subsequently, whole flowers from both WT and OE plants were harvested for analysis of volatile metabolome composition. A 500 mg sample was obtained after grinding, and then saturated sodium chloride solution and internal standard solution were added. Automatic headspace solid phase microextraction (HS-SPME) was conducted for sample extraction. The extraction condition included an extraction time of 15 min and a separation time of 5 min at 250 °C for sample analysis. The chromatographic conditions utilized high-purity helium gas as the carrier gas, with a flow rate of 1.2 mL/min and an injection port temperature of 250 degrees Celsius. The sample was injected into a splitless mode, and the programmed temperature was initially raised to 40 degrees Celsius for 3.5 min, followed by an increase to 100 degrees Celsius at a rate of 10 °C/min, then to 180 degrees Celsius at a rate of 7 °C/min, and finally to 280 degrees Celsius at a rate of 25 °C/min, held for 5 min. The mass spectrometry conditions included an ion source temperature of 230 degrees Celsius, an electron energy of 70 eV, and precise qualitative and quantitative ion scanning. Differential metabolites were identified based on fold change greater than 2 or less than 0.5.

## 3. Results

### 3.1. Gene Cloning and Structural Analysis of DcTPSb1 from D. chrysotoxum Flowers

The open reading frame (ORF) of *DcTPSb1* extended over 1797 bp and consisted of seven exons, as highlighted in Figure S2, encoding a sequence of 598 amino acids. The molecular size and isoelectric point of this protein sequence were 69.49 kDa and 5.21, respectively. Analysis of the DcTPSb1 secondary structure utilizing the SOPMA program revealed that the protein comprised 65.05% α-helix, 27.59% random coil, 3.85% extended strand, and 3.51% β-turn (Figure S3).

### 3.2. Sequence Alignment Analysis of DcTPSb1 from the D. chrysotoxum Genome

**Figure 1:**
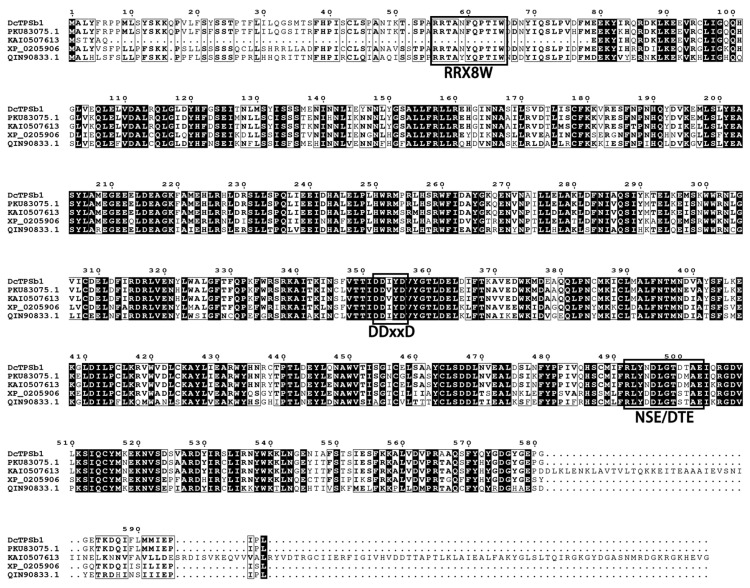
Comparison of amino acid sequence of DoTPSb1 protein in *Dendrobium chrysotoxum* with four high similarity sequences from NCBI (PKU83075.1, Alpha-terpineol synthase of *Dendrobium catenatum*; KAI0507613.1, hypothetical proteins KFK09_013739 of *Dendrobium nobile*; XP_020590622.1, terpene synthase 10-like of *Phalaenopsis equestris*; QIN90833.1, terpenoid synthase of the *Oncidium hybrid cultivar*). Meanwhile, three motifs critical for monoterpene synthesis, including two motifs rich in aspartic acid residues, DDxxD and NSE/DTE, and the arginine–tryptophan motif, RRX8W, are marked out in the above image. Completely identical sequences are marked in black, four identical amino acids are marked with boxes, and the rest of the alignments are not marked. Related detailed information is shown in Table S3.

The protein DcTPSb1 was subjected to a BLAST search in NCBI, which identified a complete alignment with KAH0460538.1 (IEQ34_011201) from the *Dendrobium chrysotoxum* genome, thereby confirming their sequence identity. Subsequent BlastP analysis revealed that DcTPSb1 exhibited significant similarity to a terpene synthase, with the highest similarity of 91% observed between DcTPSb1 and Alpha-terpineol synthase of *Dendrobium catenatum* (PKU83075.1). Additionally, similarities of 75%, 73%, and 68% were found between DcTPSb1 and the hypothetical proteins KFK09_013739 of *Dendrobium nobile* (KAI0507613.1), terpene synthase 10-like of *Phalaenopsis equestris* (XP_020590622.1), and terpenoid synthase of the *Oncidium hybrid cultivar* (QIN90833.1), respectively. Based on the high sequence identity, four proteins were selected for multiple sequence alignment. The alignment revealed that all proteins exhibited two motifs rich in aspartic acid residues, DDxxD and NSE/DTE, located at the C-terminal. Notably, except for the hypothetical protein KAI0507613.1 from *Dendrobium nobile*, all proteins contained an RRX8W (arginine–tryptophan motif) domain at the N-terminal. Previous research has highlighted the essential role of the DDxxD and NSE/DTE motifs in the binding of cofactors such as Mg^2+^ or Mn^2+^, which are critical for the cleavage of prenyl diphosphate substrates, ultimately leading to the synthesis of monoterpenes [28,29,30]. Furthermore, the RRX8W domain has been associated with the cyclization process of monoterpene synthases [31], thereby reinforcing the classification of DcTPSb1 within the TPS-b subfamily, as illustrated in Figure 1.

### 3.3. Phylogenetic Analysis of DcTPSb1 Protein

**Figure 2:**
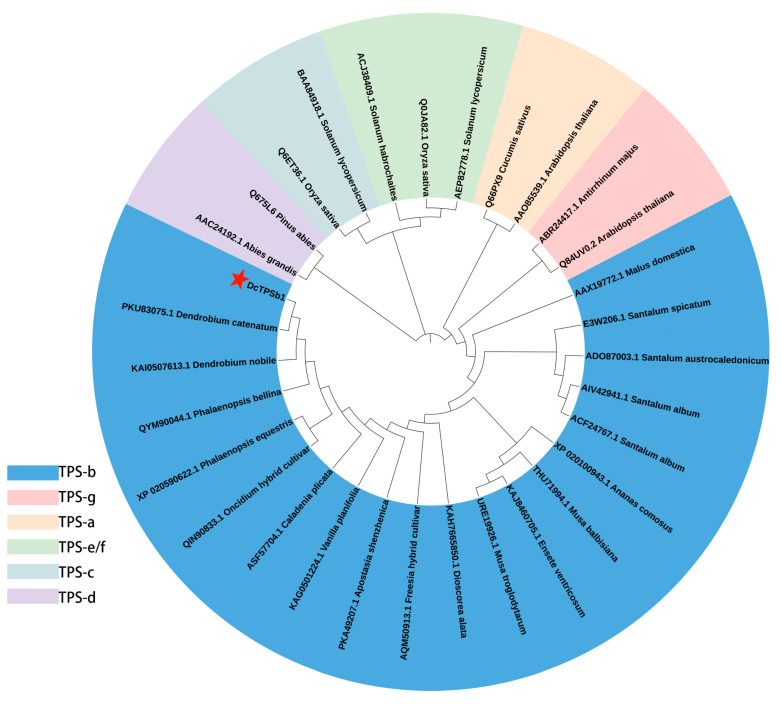
The evolutionary tree was formed based on the neighbor-joining method in the MEGA 11.0 tool. All sequences belonging to several different plants were obtained from NCBI. Seven TPS subfamilies were shown in six colors, in which DcTPSb1 protein from *D. chrysotoxum* was distributed in the largest blue area of the TPS-b family (marked with red star). All sequence information can be found in Supplementary Table S3.

The phylogenetic analysis map was generated on the basis of the results of multiple sequence comparisons of 14 homologous sequences with higher similarity from various plant species including Ananas comosus, Apostasia shenzhenica, Caladenia plicata, Dendrobium catenatum, Dendrobium nobile, Dioscorea alata, Ensete ventricosum, Freesia hybrid cultivar, Musa balbisiana, Musa troglodytarum, Oncidium hybrid cultivar, Phalaenopsis bellina, Phalaenopsis equestris, and Vanilla planifolia (Figure 2). Additionally, 16 sequences representing seven subfamilies (TPS a–g) from different species were incorporated into the analysis. The phylogenetic tree revealed six distinct clusters, with DcTPSb1 classified within the TPS-b subfamily. Notably, DcTPSb1 exhibited a higher sequence similarity to PKU83075.1 (identified as alpha-terpineol synthase) from Dendrobium catenatum and KAI0507613.1 from Dendrobium nobile. This suggests a closer genetic relationship between Dendrobium species, indicating a more significant genetic affinity within the same species.

### 3.4. Subcellular Localization of DcTPSb1

**Figure 3:**
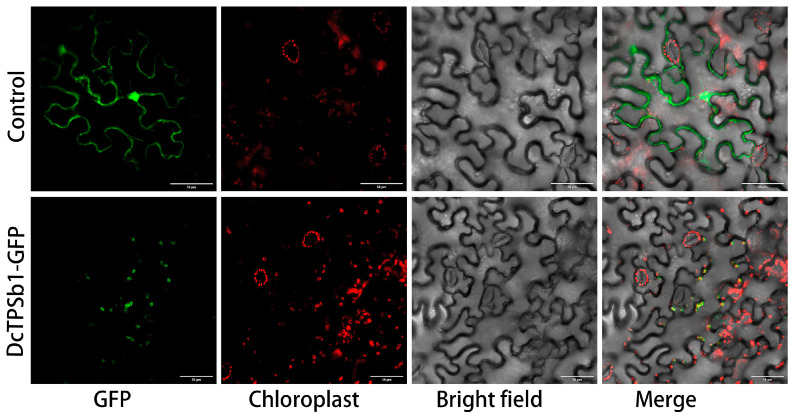
Graph of the result of an experiment on subcellular localization of DcTPSb1 from *D. chrysotoxum*. Red represents chloroplast autofluorescence; green represents GFP display fluorescence; yellow represents fluorescence of the fusion protein connecting GFP and DcTPSb1, i.e., indicating cellular localization of the DcTPSb1 protein.

Initially, the subcellular localization of DcTPSb1 was predicted using prediction tools like WoLF PSORT and pLoc-mPlant, which suggested that DcTPSb1 is located in chloroplasts and is likely a mono-TPS in the MEP-pathway rather than in the cytosolic MVA pathway. To confirm this localization, a vector containing a GFP fragment linked with the *DcTPSb1* sequence was transformed into *N. benthamiana* leaves. Subsequently, confocal laser scanning microscopy was used to visualize yellow fluorescence signals. The resulting images clearly show that DcTPSb1 was indeed localized in chloroplasts, providing experimental validation of the initial prediction (Figure 3).

### 3.5. Expression Mode Analysis of DcTPSb1 in Different Tissues from D. chrysotoxum

**Figure 4:**
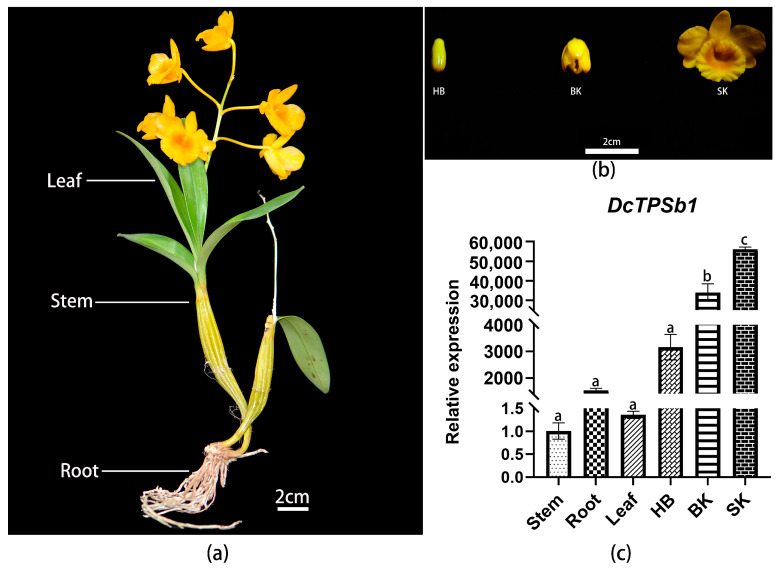
Different tissues of *D. chrysotoxum*, include leaf, stem, root (**a**) and three developmental stages of flowers (**b**). (**c**) Expression levels of *DcTPSb1* in different *D. chrysotoxum* tissues 18S served as the internal standard. HB, bud stage; BK, half blooming stage; SK, full blooming stage. Each bar represents the mean ± standard error of three independent biological replicates. The one-way ANOVA was performed to identify the differences among experimental groups, and different lowercase letters indicated significant differences *p* < 0.001.

The expression patterns of genes across various tissues are essential for understanding their regulatory mechanisms. In higher plants, related TPS genes have tissue specificity, occurring in tissues such as flowers, leaves, and roots, similar to the temporal and spatial specificity of volatile terpene release. Therefore, samples from different tissues of *Dendrobium chrysotoxum* were collected to investigate the expression differences in DcTPSb1 across four common plant tissues and to explore its expression profile. Specifically, flower tissue samples were collected at three different periods, spanning from the budding to half opening period to the blooming period. Subsequently, a quantitative PCR experiment (QPCR) was employed to quantify the expression level differences in DcTPSb1 across these different tissues and developmental stages. The findings revealed that *DcTPSb1* displayed extremely low expression levels in stems and leaves, lower expression in roots (Figure 4a), and the highest expression in flowers (Figure 4b). Moreover, as the flowers progressed through their developmental stages, the expression levels of *DcTPSb1* mRNA exhibited fluctuations. Specifically, *DcTPSb1* was minimally expressed during the bud stage, experienced a significant increase in expression during the semi-open flowers stage, and reached its peak expression level in fully open flowers (Figure 4c). These outcomes indicate that the floral terpenes regulated by *DcTPSb1* are predominantly emitted during the flowering stage of *D. chrysotoxum*, with a notable release occurring during the fully open flower stage.

### 3.6. Prokaryotic Protein Expression and In Vitro Functional Validation of DcTPSb1

**Figure 5:**
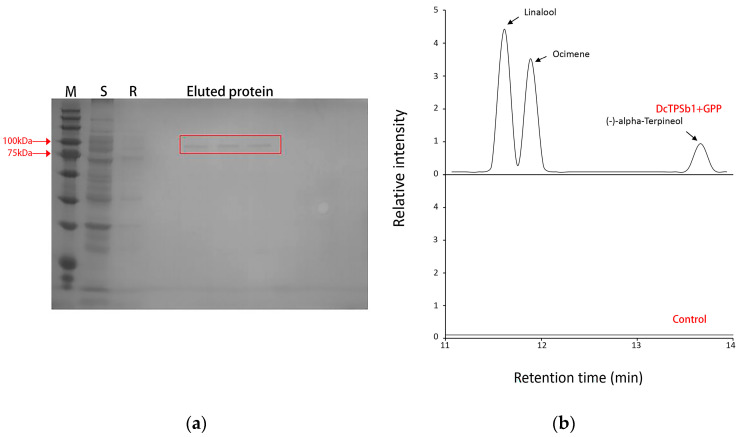
Functional characterization of DcTPSb1. (**a**) Purification of DcTPSb1 recombinant protein. M, marker; S, supernatant; R, rinse solution. The red frame indicates purified target protein; Eluted protein represents GST-DcTPSb1 protein with 89.2 KDa. (**b**) Gas chromatograms of three monoterpenes generated by DcTPSb1 when GPP (Geranyl pyrophosphate) was used as a substrate. Control added high-temperature inactivated DcTPSb1 protein, in which no products were generated.

Following expression, the proteins were purified, where supernatant, rinse solution, and eluent were separated out showcasing a single band on an SDS-PAGE gel, matching the expected size of DcTPSb1 (Figure 5a). To further verify its in vitro function, GPP was used as a substrate for incubation with recombinant DcTPSb1 protein. GC-MS detection of the resulting products revealed that the recombinant DcTPSb1 protein yielded three monoterpenes, linalool, ocimene, and (-)-α-pinacol (Figure 5b). Notably, the three monoterpenes aforementioned were undetected in a heat-inactivated protein mixture, thereby confirming the specificity of DcTPSb1. These findings collectively indicated that DcTPSb1 from *D. chrysotoxum* exhibits the capability to catalyze the formation of linalool, ocimene, and (-)-alpha-Terpineol. Consequently, due to its ability to produce multiple terpenes, DcTPSb1 was appropriately classified as a multi-product terpene synthase.

### 3.7. Discernment of Cis-Acting Elements in the Promoter Region of DcTPSb1

**Figure 6:**
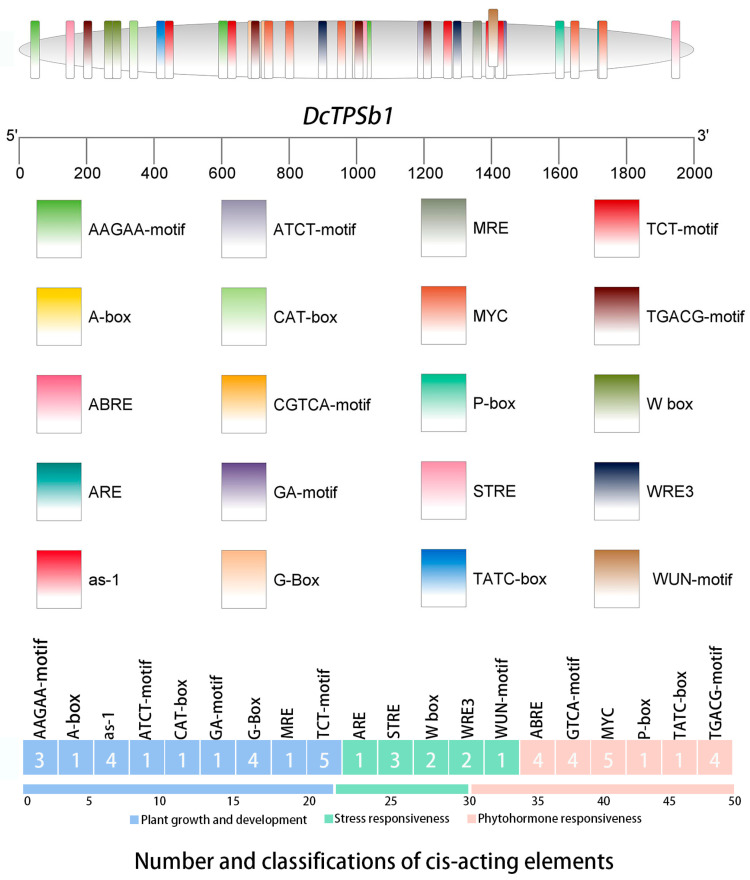
The distributions, categories, and numbers of twenty cis-acting elements in 2000 bp upstream region of the initiation codon (ATG) of *DcTPSb1* in *D. chrysotoxum*. The above figure represents a wide distribution throughout the entire region of 20 cis-acting elements (represented by 20 types of blocks, respectively) distributed throughout the entire region with three categories in three colors. The following figure shows 20 types of cis-acting components belonging to three categories, represented by three colors. The detailed distribution of motifs is shown in Table S4.

The promoter region of *DcTPSb1* contains a wide variety of cis-acting elements commonly found in promoter sequences. These cis-acting elements play important roles in modulating the biological function of genes. According to their functions related to stress, phytohormones, plant growth and development, these elements are often categorized into three categories in Figure 6. Within subgroup related to plant growth and development (21/49), a total of nine types of cis-elements involved in endosperm expression (AAGAA-motif), shoot expression (As-1 element), as well as meristem expression of root and apical (A-box, CAT-box), light responsiveness (ATCT-motif, GA-motif, G-Box, MRE, and TCT-motif) were observed, with the TCT-motif being the most prevalent at 24%. In the stress responsiveness category (9/49), diverse elements linked to stress (STRE, 33%), anaerobic induction (ARE, 11%), and wounding (W box, WRE3, and WUN-motif, each at 22%, 22%, and 11%, respectively) responsiveness were identified. The majority of cis-elements (19/49) fell under the phytohormone responsiveness category, responding to abscisic acid (ABRE), MeJA (MYC, CGTCA-motif, and TGACG-motif), and gibberellin (P-box and TATC-box). Particularly notable was the prevalence of MeJA-responsiveness elements, comprising 68% of the hormone-related cis-elements. Based on these findings, it is suggested that *DcTPSb1* could potentially be regulated by MeJA or light, inducing or repressing its expression.

### 3.8. Analysis of Volatile Metabolite Differences Between Tobacco Overexpressing DcTPSb1 and Wild-Type Using GC-MS

**Figure 7:**
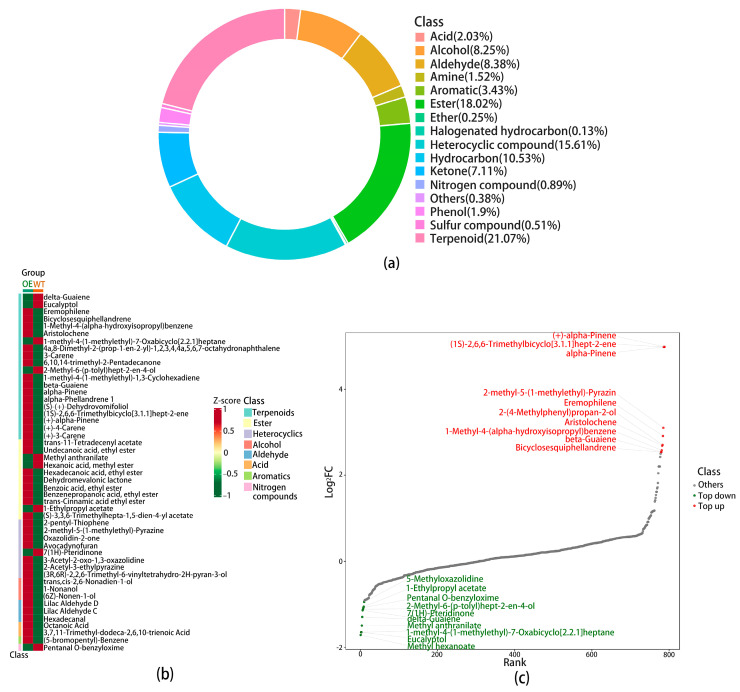
Analysis of volatile metabolome results of GC-MS analysis between DcTPSb1-overexpressed tobacco and wild type. (**a**) Ring diagram of metabolite class composition. Each color represents a metabolite category, and the area of the color block indicates the proportion of that category; (**b**) Heat map of differential metabolite clustering. On the left side of the figure is the sample clustering line, and on the top is the sample clustering line. Class is the first level classification of substances. Different colors are the colors filled with different values obtained after standardizing the relative content. Red represents high content, and green represents low content; (**c**) Dynamic distribution of differences in levels of differential metabolites. Each point represents a substance, with green points representing the top ten substances ranked downwards and red points representing the top ten substances ranked upwards.

Compared to the wild type, there were no floral phenotypic changes in the transgenic tobacco plants. Subjecting blooming flowers from empty and overexpressed plants to GC–MS analyses revealed the presence of terpenoid VOCs in *N. benthamiana*, accounting for 21.07% of the total VOCs (Figure 7a), a notably higher percentage than other compound types, including 166 terpenoid varieties. The substance with significant changes is shown in Figure 7b. Among the significantly altered metabolites in overexpressed tobacco, terpenes were the most diverse and varied. DcTPSb1, members of the TPS-b subfamily known as monoterpene synthases, likely played a crucial role in regulating the changes in the monoterpene level. Figure 7c displays the top 10 upregulated and top 10 downregulated substances. Noteworthy changes were observed in the relative content of three monoterpenes-α-Pinene, (1S)-(-)-alpha-Pinene, and (1R)-(+)-α-Pinene-with significantly increased levels among the differential metabolites.

## 4. Discussion

Based on previous studies, the TPS family is typically organized into seven or eight subfamilies, including TPS-a, -b, -c, -d, -e/f or -e/-f, -g, and -h, distinguished by their sequence’s heterogeneity and phylogenetic relationships [32]. The TPS-e/f subfamily, both classified as diterpene synthases, are prevalent in vascular plants, with TPS-f identified as a derivative of TPS-e and often grouped with it. TPS-c, a diterpene synthase, is distributed in both gymnosperms and angiosperms. The TPS-d subfamily, which is specific to gymnosperms, is responsible for the synthesis of a complex mixture of monoterpenes, sesquiterpenes, and diterpenes. Conversely, TPS-h is exclusively detected in stone pines (*Pterocarpus indica*). Among these subfamilies, TPS-a primarily encodes for sesquiterpene synthase in monocotyledonous plants and diterpene synthase in dicotyledonous plants [33]. Predominantly found in angiosperms, TPS-b and TPS-g are responsible for monoterpene synthesis. Notably, TPS-b features the essential metal-binding domains C-terminal DDxxD, NSE/DTE, and the N-terminal RRX8W motif, crucial for binding divalent metal ions such as Mg or Mn, all integral to monoterpene synthesis. In contrast, TPS-g does not contain the RRX8W motif [34]. In this study, the DcTPSb1 from *D. chrysotoxum*, which consisted of 598 amino acids and possessed all three aforementioned motifs, was unequivocally classified within the TPS-b family. Moreover, experimental findings indicate its localization in the chloroplast, consistent with the majority of TPS-b family members.

Phylogenetic analysis revealed that DcTPSb1 was clustered with alpha-terpineol synthase from *D. catenatum* (PKU83075.1) and from *D. nobile* (KAI0507613.1), indicating a closer genetic relation among species within *Dendrobium* genus. This observation is consistent with the general principle that plants within the same genus are more closely related. However, discrepancies were noted in the examination of gene structure and amino acid alignment. Specifically, KAI0507613.1 from *D. nobile* was found to lack the RRX8W domain, indicating that it does not belong to the TPS-b family, in contrast to the other four proteins utilized for sequence alignment, all of which contained the three requisite domains. This observation supports Bohlmann’s hypothesis that TPSs from closely related plant species tend to cluster together more prominently than enzymes with similar functions, posing challenges for predicting substrates/products based solely on sequence similarities [35]. The intricacies of studying plant affinities underscore the complexity of the process, wherein outcomes may differ based on the chosen sequences and methods. Variations in results may arise even when using different approaches on the same sequences. Therefore, a comprehensive strategy that integrates multiple methodologies is essential for achieving a more robust determination, underscoring the necessity for further exploration in this domain.

Transcription factors (TFs) are integral to the regulatory mechanisms governing gene expression, as they recognize and bind to cis-acting elements. In response to adverse environmental conditions, specific transcription factors become activated and interact with cis-acting elements located in the promoter region of stress-related genes, thereby facilitating their expression [36]. In the promoter of *DcTPSb1*, various cis-acting elements associated with different biological roles were identified, with a notable prevalence of elements linked to light responsiveness and MeJA signaling. MeJA is known to enhance the expression of TPS genes, which in turn increase the production of monoterpene volatiles. Consequently, MeJA treatment is likely to induce the expression of *DcTPSb1*, as the G-box is present in its promoter. This expression is modulated through interactions between G-box and CGTCA or MYC motifs in the MeJA signal transduction pathway [37], as well as through interactions with transcription factors such as OsMYC2 in rice or TcbZIP60 in *Tanacetum cinerariifolium* [38,39]. Additionally, light conditions may serve as either stimulants or inhibitors, impacting the expression of *DcTPSb1*.

The temporal and spatial specificity of terpene emission and expression patterns of TPS genes in higher plants have been well documented [40,41]. For instance, in *P. bellina*, the specific expression of *PbGDPS*, which is involved in the synthesis of geraniol and linalool, is predominantly observed in flowers, with peak expression occurring after four days of flowering [42]. The expression levels of the *LIS1* gene (monoterpenoid synthase producing linalool after translation) in the petals of *Osmanthus fragrans* D‘angui’ are positively correlated with the release of linalool [43]. Notably, the release of monoterpenes is found to occur mainly at the onset of anthesis in three species belonging to the genus Maxillaria, and family *Maxillariinae*, and the site of production is sepal [44]. In the case of *D. chrysotoxum*, the expression of *DcTPSb1* is significantly higher in flowers, particularly during the flowering stage, in comparison to leaves, stems, and roots, indicating a stringent regulation of terpenoid production. While volatile compound biosynthesis and emission are typically developmentally regulated, with peak expression in early developmental stages like young leaves, unfertilized flowers, and unripe fruits [45], in *D. chrysotoxum*, the expression of *DcTPSb1* escalates continuously from the budding stage, reaching its highest levels at the peak of flowering. This observation may suggest that GPP accumulates substantially in fully boom flowers, leading to the production of abundant terpenoid compounds that are subsequently released. Moreover, environmental factors such as light, temperature, and circadian rhythm are known to influence volatile aroma emissions [11,46], though this inference requires further investigation.

DcTPSb1 was characterized as a multi-product terpene synthase associated with the synthesis of a monoterpene, linalool, ocimene, and (-)-alpha-Terpineol, like other multi-product enzymes, such as FhTPS4, FhTPS6, and FhTPS7 found in *Freesia x hybrida* [47]. In contrast, it differs from DoTPS10, DoGES1, and CmTPS2, which encodes a single product in *Dendrobium officinale* and *Clivia miniata* [15,16,17,48]. However, these three products are completely different from the seven aromatic monoterpenes involved in the transcriptional metabolic network. This phenomenon is consistent with our research on DlTPSb1/DlTPSb2 proteins in *D. loddigesii* [4], and their catalytic products are not related terpenes in their transcriptional metabolic network. The transcriptional metabolic regulatory network predicts the regulatory role between genes, transcription factors, and terpene compounds. But it may not necessarily mean that there is a direct regulatory relationship between these three. Generally, genes can be screened for research based on this, but the specific functions and regulation mechanisms of genes need further experimental verification. In experiments involving the overexpression of *DcTPSb1* in *N. benthamiana*, the anticipated increase in the production of the three monoterpenes products encoded by the DcTPSb1 protein was not observed. Instead, the relative contents of the terpenoids were up-regulated, consistent with the expected outcomes. Notably, among the terpenoids, α-pinene, (1S)-(-)-α-pinene, and (1R)-(+)-α-pinene exhibited significant up-regulation, while the three monoterpenes produced by DcTPSb1 remained mostly unchanged. This finding contrasts with LiTPS2 from *Lilium longiflorum ‘Siberia’* in transgenic tobacco plants, where the overexpression facilitates the release of its product, linalool [49]. However, it aligns with the results observed for PbTPS5 and PbTPS10 (*Phalaenopsis bellina*) ectopically overexpressed in *N. tabacum*, which did not produce linalool or geraniol [14]. These observations suggest that the terpene synthesis pathway may undergo different processes in vivo and in vitro. As a model organism, tobacco may exhibit significant differences from the actual terpene synthase source plants, potentially lacking other related genes necessary for terpene biosynthesis, thus impeding the desired product’s production. The observed disparities in terpene compound changes may arise from various factors, underscoring the necessity for further experimental investigations to confirm these findings while controlling for potential confounding variables. Moreover, the presence of transcription factors, enzymes, and other essential factors is crucial for the biosynthesis of terpene, which may be absent in *N. tabacum* or other non-native plants. Conversely, the presence of repressors in non-native plants could inhibit terpene biosynthesis, even if the required transcription factors and key enzymes are provided.

## 5. Conclusions

In the present study, a preliminary analysis of *DcTPSb1* in *Dendrobium chrysotoxum* was conducted, encompassing examinations of the exonic intron structure distribution, conserved motifs, subcellular localization, spatiotemporal expression pattern, cis-acting elements, phylogenetic analysis, in vitro functional validation, and gene overexpression. *DcTPSb1* was found to exhibit differential expression patterns throughout three floral phases and various tissues, underscoring its role in regulating monocotyledon biosynthesis in flowers. DcTPSb1 localized in chloroplasts, was a member of the TPS-b subfamily, closely related to homologous plants. Functionally, DcTPSb1 could convert geranyl diphosphate (GPP) to linalool, ocimene, and (-)-α-pinitol in vitro. This study offers insights into the regulatory mechanisms of TPS genes in *Dendrobium* spp. and Orchidaceae, laying a foundation for future investigations and potential modifications of floral scents in these plants.

## Data Availability

Data are contained within the article and Supplementary Materials.

## Supplementary Materials

The following supporting information can be downloaded at: https://www.mdpi.com/article/10.3390/cimb47010025/s1, Figure S1: Agarose gel electrophoresis of cDNA amplification of the *DcTPSb1* gene; Figure S2: Sequence analysis of *DcTPSb1* genome in *Dendrobium chrysotoxum*, including 6 introns, 7 exons and UTR sequences at both ends; Figure S3: Second class structure of DcTPSb1; Figure S4: Specific distribution of three important motifs commonly distributed in TPS proteins in Figure 1; Figure S5: Validation of genetically modified tobacco; Table S1: DNA sequence of *DcTPSb1* and protein sequence of DCTPSb1 from the *D. chrysotoxum*; Table S2: Information on primers used for sequence amplification and validation in this paper; Table S3: Information about proteins with relatively similar homology to the target protein and other proteins known to belong to the TPS subfamily used in Figure 1 and Figure 2; Table S4: Cis-acting elements in the promoter region of *DcTPSb1* gene; Table S5: The upstream promoter 2 kb of *DcTPSb1* gene.
