# Supplementary material for: Expression Analysis and Functional Validation of DcTPSb1 in Terpene Synthesis of Dendrobium chrysotoxum
Source: Curr Issues Mol Biol. 2025 Jan 3;47(1):25. doi: 10.3390/cimb47010025 (PMC11763578; doi:10.3390/cimb47010025)
Supplement: Supplementary file 1 [file cimb-47-00025-s001.zip › cimb-3332048-supplementary figures.pdf]

SUPPLEMENTARY MATERIALS

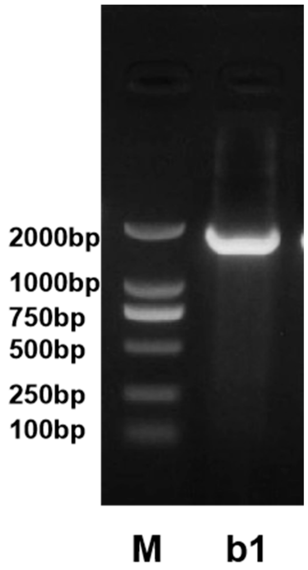

Figure S1. Agarose gel electrophoresis of cDNA amplification of the *DcTPSb1* gene. M, marker; b1, cDNA of *DcTPSb1*, with a length of 1797bp.

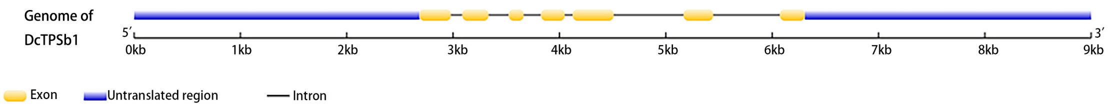

Figure S2. Sequence analysis of *DcTPSb1* genome in *Dendrobium chrysotoxum.* The sequence included 6 introns (gray line distributed between exons), 7 exons(yellow rectangle), and UTR (Untranslated Region, blue rectangle) sequences at both ends.

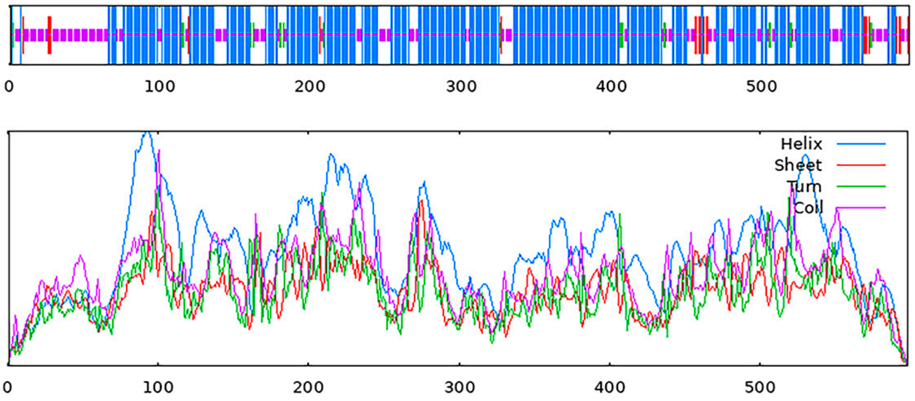

Figure S3. Second-class structure of DcTPSb1, which was composed of 65.05% α-helix(389 amino acids), 27.59% random coil(165 amino acids), 3.85% extended strand(23 amino acids), and 3.51% β-turn(21 amino acids). The four structures are shown in the blue, purple, red, and green colors, respectively, in the figure.

Name	Start	p-value		Sites	
DcTPSb1	313	1.17e-63	NLGVICDELD	FIRDRLVENYLWALGFTFQPKFWRSRKAITKINSFVTTIDDIYDVYGTLD	ELDIFTKAVE
PKU83075.1	313	1.38e-63	NLGVLCDELD	FIRDRLVENHLWALGFTFQPKFWRSRKAITKINCLVTTIDDVYDVYGTLD	ELEIFTNAVE
KAI0507613.1	241	5.04e-63	NLGVLCDELD	FIRDRLVENHLWALGFTFQPKFWRSRKAITKINSLVTTIDDVYDVYGTLD	ELEIFTNVVE
XP_020590622.1	313	2.50e-56	NLGLVCDELN	FARDRLVENYLMALGFTFQPEFWRIRKAITKLNSFVTTIDDIYDVYGTLD	ELKLFTNAVE
QIN90833.1	313	2.52e-54	NCGLICEELN	FIRDRLVENYLWSIGFNCQPEFGRSRKAIAKINCLVTTIDDIYDVYGTLD	ELKLFTNAIK

Name	Start	p-value		Sites	
DcTPSb1	479	1.72e-64	LNVEALDSLN	FYPPIVQHSCMIFRLYNDLGTDTAEIQRGDVLKSIQCYMKEKNVSDSVAR	DYIRSLIRNY
PKU83075.1	479	1.34e-63	LNVEALDSIK	FYPPIVQHSCMIFRLYNDLGTDMAEIQRGDVLKSIQCYMNEKNVSDSAAR	DYIRCLIRNY
KAI0507613.1	407	6.69e-63	LNVEALDSIN	FYPPIVQHSCMIFRLYNDLGTDMAEIKRGDVLKSIQCYMNEKNVSDSAAR	DYIRCLIRNY
QIN90833.1	479	9.95e-58	LTIEALKSFE	FYPPIFRHSCMLFRLYDDLGTSTAEIKRGDVPKSIQCYMKEKNVSEPIAR	DYIRCLIKKY
XP_020590622.1	479	1.15e-52	LTSEALNKLE	FYPSVARHSSMLFRLYDDLGTSTAEIQRGDVSKSIQCYMREKNVSEPFAR	DHIRYLIRNY

Name	Start	p-value		Sites	
DcTPSb1	50	7.90e-36	PISCLSPANT	KTSPARRTANFQPTIWDDNYIQSLPVD	FMEEKYIRQR
XP_020590622.1	50	7.09e-34	ICCLSTANAV	SSTPARRTANYQPTIWDDNYIQSLPID	FMEEKYIHRR
PKU83075.1	50	1.67e-33	PISCLSTANT	KTSPARRTANFQPTIWDDNYIQSLPVH	FMEEKYKHQR
QIN90833.1	50	1.55e-29	IRCLQIAAQI	SSSPPRRTAYYQPTIWNDDYIQSLPID	FMDEKYVYER

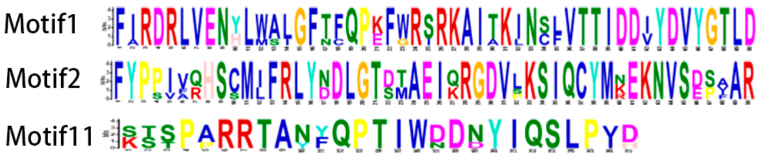

Figure S4. Specific distribution of three important motifs commonly distributed in TPS proteins in Figure1. Motif1/2/11 represents the distribution of DDXXD, NSE/DTE, and RRX8W, respectively. Color-coded amino acid regions indicate the specific positions of motifs.

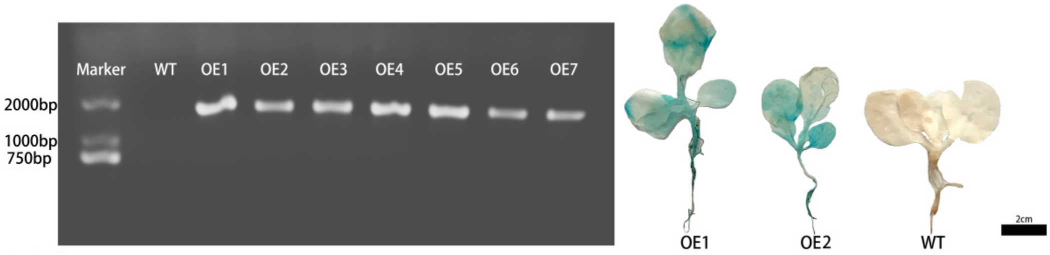

Figure S5. Validation of genetically modified tobacco. The left and right images, respectively, represent PCR experiments and GUS(β-glucuronidase) staining. OE1-7, 7 *DcTPSb1*-overexpressed tobacco; WT, wild-type tobacco. PCR products with a length of 1797bp generated in transgenic benthamiana plants ,while no band was generated in WT. After GUS(β-glucuronidase) staining, the overexpressed tobacco appears blue, while the wild-type tobacco appears white.
